# Synthesis and Characterization of Biodegradable Polyurethane for Hypopharyngeal Tissue Engineering

**DOI:** 10.1155/2015/871202

**Published:** 2015-03-08

**Authors:** Zhisen Shen, Dakai Lu, Qun Li, Zongyong Zhang, Yabin Zhu

**Affiliations:** ^1^Lihuili Hospital, Ningbo University, 818 Fenghua Road, Ningbo 315211, China; ^2^The Medical School, Ningbo University, 818 Fenghua Road, Ningbo 315211, China; ^3^School of Chemical Engineering, Ningbo University of Technology, 201 Fenghua Road, Ningbo 315211, China

## Abstract

Biodegradable crosslinked polyurethane (cPU) was synthesized using polyethylene glycol (PEG), L-lactide (L-LA), and hexamethylene diisocyanate (HDI), with iron acetylacetonate (Fe(acac)_3_) as the catalyst and PEG as the extender. Chemical components of the obtained polymers were characterized by FTIR spectroscopy, ^1^H NMR spectra, and Gel Permeation Chromatography (GPC). The thermodynamic properties, mechanical behaviors, surface hydrophilicity, degradability, and cytotoxicity were tested via differential scanning calorimetry (DSC), tensile tests, contact angle measurements, and cell culture. The results show that the synthesized cPU possessed good flexibility with quite low glass transition temperature (*T*_*g*_, −22°C) and good wettability. Water uptake measured as high as 229.7 ± 18.7%. These properties make cPU a good candidate material for engineering soft tissues such as the hypopharynx. *In vitro* and *in vivo* tests showed that cPU has the ability to support the growth of human hypopharyngeal fibroblasts and angiogenesis was observed around cPU after it was implanted subcutaneously in SD rats.

## 1. Introduction

Recently, the rate of pharyngeal cancer has been increasing year after year due to alcohol and tobacco abuse [1]. In the literature, 60–80% of hypopharyngeal head and neck tumor patients were reported to have developed ipsilateral lymph node metastasis upon presentation of clinical symptoms, leading an overall five-year survival rate of 33.4% [2, 3]. Surgical removal of the apparent tumor is still an important clinical treatment, although evidence suggests that chemotherapy and radiotherapy can help to control tumor growth and promote patient survival rates [4]. Tumor resection results in tissue defects, which inevitably leads to chronic health problems including hindered breathing, swallowing, vocalizing, and possible mental illness. Clinical surgery is usually performed using jejunal free flap, lateral thigh flap, or pectoralis major myocutaneous flap as the substitutes. However, due to the complex structure of the throat, these substitutes cannot restore pharyngeal function and often result in flap necrosis, infection, fistula, or stenosis [5–8]. Therefore, it is necessary to find effective and safe substitutes for laryngeal reconstruction.

The rapid development of tissue engineering brings new possibilities for laryngeal and hypopharyngeal reconstruction. Biodegradable materials with good mechanical strength, elasticity, and nontoxicity are good potential candidates as biological substitutes. Poly(lactic acid) (PLA) is a promising material which has been approved by the Food and Drug Administration (FDA) for medical applications. However, there are some drawbacks with the use of PLA as a matrix for hypopharyngeal tissue. For example, it is a stiff polymer with a high glass transition temperature (*T*_*g*_) of 65°C, which does not meet the requirements of laryngeal tissue growth.

Polyurethane is a widely used material in tissue engineering because of its good mechanical properties, biocompatibility, and biodegradability [9, 10]. It is usually synthesized via a crosslinking reaction between diisocyanate and polyhydric hydroxy with polyol or diamine as the extender. Some components such as caprolactone, poly(ethyleneoxide), lactic acid, and ricinoleic acid have been experimented with in the production of thermoplastic and biodegradable polyurethanes [11–14]. In our previous work, we studied the catalysis of aliphatic ester polymerization by low toxicity iron compounds, instead of the stannum compounds used commonly in research or/and industry [15]. Using this approach, we synthesized a biocompatible and biodegradable copolymer with *T*_*g*_ of 5.6°C from monomers of L-lactide, poly(ethylene glycol) (PEG), and NIPAAm [16]. However, the previously published polymer was too rigid to be used in reconstruction of soft tissues like the hypopharynx. In this study, we have produced a material with lower *T*_*g*_, better strength, and superior biodegradability using reactions between L-lactide, PEG, and hexamethylene diisocyanate (HDI). The frequency and chemistry of the reaction were characterized by Fourier transform infrared spectroscopy (FTIR), Hydrogen-1 nuclear magnetic resonance (^1^H NMR) spectra, and Gel Permeation Chromatography (GPC). The novel material's mechanical property, hydrophilicity, degradability, cytocompatibility, and* in vivo* biocompatibility were evaluated. We believe that the material presented in this paper will be a good substitute for hypopharyngeal tissue engineering.

## 2. Materials and Methods

### 2.1. Materials

PEG (Mn 2000), dichloromethane (CH_2_Cl_2_), ethanol, and ethyl acetate were purchased from Sinopharm Chemical Reagent Co., Ltd. L-lactide (L-LA) was supplied by Jinan Daigang Biological Material Company. It was recrystallized three times using ethyl acetate as the solvent prior to reaction. Iron (III) acetylacetonate (Fe(acac)_3_) and HDI were purchased from Aladdin Reagent Co., Ltd., China.

Trypsin (1 : 250, GNM) was purchased from Beijing Genosys Scientific Co., China. Mouse anti-vimentin and FITC-conjugated goat anti-mouse IgG were from Wuhan Boster Bio-Engineering Co., Ltd. IRDye 680RD goat anti-mouse IgG (H+L) was purchased from LI-COR Biosciences, USA. All cell culture reagents were purchased from HyClone unless otherwise specified. All chemical reagents used for Western blotting were purchased from Beyotime Institute of Biotech, Jiangsu, China. Phosphate buffer saline (PBS, pH7.4) used in cell culture was sterilized prior to use. Sprague-Dawley (SD) male rats were obtained from the Experimental Animal Center of Ningbo University.

### 2.2. Synthesis of PLA-PEG-PLA Prepolymer (PLEG)

The prepolymer PLEG was synthesized using L-LA and PEG (Mn 2000) as monomers and Fe(acac)_3_ as the catalyst, as per our previous protocol [12]. Briefly, predetermined amounts of PEG and L-LA were added into a polymerization tube with Fe(acac)_3_ (0.5%, weight ratio to total reactants, resolved in CH_2_Cl_2_) as the catalyst. After degassing for 2 h at 60°C, the tube was sealed under vacuum and placed entirely into an oil bath and the bath temperature was maintained at 130°C for 20 h with stirring. The polymerization tube was then taken out of the oil bath and cooled to room temperature. Ten milliliters CH_2_Cl_2_ was added to dissolve the product. After precipitation in ethanol at 0°C, the three-block PLA-PEG-PLA prepolymer (PLEG) with different ratios of monomers was obtained (Table 1). The PLEG was subsequently dried in a vacuum oven and kept in the dryer for the next reaction.

### 2.3. Preparation of Crosslinked Polyurethane

Prepolymer PLEG, PEG, and HDI (molar ratio, 1.65 : 10 : 2.9) were mixed and heated to 80°C for 8 h under nitrogen protection to allow crosslinking to occur. PLEG and PEG were separately dehydrated via degassing for 1 h at 110°C prior to the crosslinking reaction. A shallow yellow sticky solution was formed at the end of the reaction. It was dissolved in CH_2_Cl_2_ to produce a 50% (v : v) solution, which was cast onto a polydimethylsiloxane (PDMS) film and dried in an oven at 60°C overnight to yield a flat, approximately 1.0 mm thick cPU membrane. PDMS was used as the substrate as the synthesized cPU membrane could easily be peeled off.

### 2.4. Characterization of Prepolymer and Crosslinked Polymer

#### 2.4.1. PLEG Prepolymer

FTIR spectra were recorded using an FTIR instrument (Digilab FTS 3100, USA) with 4 cm^−1^ resolution and a measuring period of 500–4000 cm^−1^. The reaction chemistry of prepolymer PLEG was confirmed via ^1^H NMR measurement. PLEG sample was dissolved in CDCl_3_ and tested on a NMR spectrometer (Bruker Avance, 400 MHz, Switzerland). The molecular weight (Mn and Mw) and molecular weight distribution (Mw/Mn) were determined by GPC (Polymer Laboratories PL-GPC 50 plus, England) using polystyrene as the standard. The analysis was performed at 40°C using tetrahydrofuran (THF) as the eluent at a flow rate of 1.0 mL/min.

#### 2.4.2. Structure and Characteristics of cPU

The thermal properties of cPU were tested by differential scanning calorimetry (DSC, Pyris Diamond, USA) under nitrogen. The first heating was from 25 to 100°C at a rate of 20°C/min with 1 min station to clear the thermal history; then the sample was cooled down to −50°C at a rate of 10°C/min. The second heating started from −50°C and increased to 100°C at a rate of 20°C/min. The glass transition temperature (*T*_*g*_) and melting point (*T*_*m*_) values were taken from the second round of heating.

The static contact angles of cPU were surveyed on a surface tension-contact angle meter (DIGIDROP, GBX, France) and the droplet dynamic contact angle was tested on Dataphysics OCA20 (Germany) at ambient humidity and temperature. A drop of deionized water was approximately 1.0 *μ*L in volume. The contact angle values of samples were averaged from three different locations and expressed as mean ± standard deviation (SD).

Mechanical properties of cPU were tested with a tensile tester (Instron 3366, USA) using a linear deformation rate of 10 mm/min at 25°C. Dumbell-shaped polyurethane membranes with a gauge length of 30 mm and cross-sectional area of 0.2–0.3 mm × 1 mm were used. Larynx tissue was obtained from Lihuili hospital (Ningbo, China), under agreement of laryngeal carcinoma patients. It was tested and used as references to compare between the synthesized materials and the native tissue. Three repeats were performed for each sample.

Water uptake was tested and calculated as follows: *R*(%) = [(*W*_1_ − *W*_0_)/*W*_0_] × 100%, where *W*_0_ was the original weight and *W*_1_ was the weight of cPU after being dipped in deionized water for 10 h at room temperature. Three repeats were performed for cPU.

### 2.5. Degradation Test

Samples were cut into pieces 40 × 40 × 1 mm in size (initial weight *W*_0_) and incubated for 1, 5, 10, 20, 40, or 80 d in PBS (pH = 7.4) supplemented with 100 U/mL penicillin-streptomycin at 37°C. After rinsing in water and drying in a vacuum oven, samples weights (*W*_*l*_) were measured on an electronic analytical balance (Sartorius BS 224s, Germany). The percentage of weight loss was calculated using the following formula:

(1) $$\begin{array}{c} \text{Weight}\;\text{loss}\;\left(\%\right)=\frac{W_{0}-W_{l}}{W_{0}}\times 100\%. \end{array}$$

*W*_0_ represents the initial weight of the sample (mg) and *W*_*l*_ represents the measured weight (mg) of the same sample after different degradation times. The degradation tests were performed in triplicate for each time point.

### 2.6. Primary Human Hypopharyngeal Fibroblast Culture

Human fibroblasts were obtained from patient hypopharynx connective tissue (sample from Lihuili Hospital in Ningbo, China, under agreement of a pharyngeal carcinoma patient). The tissue sample was rinsed well in sterile PBS containing antibiotics (1000 U/mL penicillin and 1000 *μ*g/mL streptomycin sulfate) and sterilized in 75% ethanol for 5 s and in 1000 ppm NaClO solution with PBS washing between steps. After that, the tissue was cut into cubes which were approximately 1 × 1 × 1 mm in dimension and attached to a culture flask (Corning, USA) containing a small amount of culture medium, Dulbecco's Modified Eagle's Medium (DMEM), fetal bovine serum (FBS, 10%), penicillin (50 IU/mL), and streptomycin (50 IU/mL). The culture medium was amended to cover the tissue at the next day. After several days, fibroblasts extended from the tissue cubes and attached to the culture plate. These primary fibroblasts were collected and subcultured for passaging.

The cells with passage 2–4 were seeded on cPU membrane at the density of 4 × 10^4^ cells/mL. cPU membrane was cut and prelaid on culture wells (96-well culture plate) with tissue culture polystyrene (TCPS) (Corning, USA) as the positive reference. The culture was incubated in medium consisting of FBS (10%, v/v) and DMEM, supplemented with penicillin (50 IU/mL) and streptomycin (50 IU/mL) in a humidified air of 5% CO_2_ at 37°C. The culture medium was changed every 2 d. After incubation for some time, cells were fixed with 2.5% glutaraldehyde for H&E and immunofluorescent staining.

### 2.7. Hematoxylin and Eosin (H&E) Staining

H&E staining was used to help better visualize the density and morphology of fibroblasts on the cPU membrane due to the opacity of the membrane. The cultures were rinsed three times with PBS and fixed with 4% paraformaldehyde (Sigma, USA) for 30 minutes at room temperature. The cultures were rinsed thrice with water to remove residual paraformaldehyde and phosphate salts. Subsequently, they were immersed in hematoxylin solution (Beijing Solarbio Science & Technology, China) for 30 min to stain cell nuclei, followed by dipping in 1% hydrochloric acid/alcohol solution for 30 s to remove the excessive hematoxylin and washing with running water for 5 min. Finally, the samples were immersed in 0.5% eosin solution (Beijing Solarbio Science & Technology, China) for 1 min to stain the cytoplasm. H&E staining images were taken under light microscopy (Model CX40, Olympus, Japan).

### 2.8. Immunofluorescent Staining

The fibroblasts seeded on cPU membranes were fixed in 4% paraformaldehyde for 10 min at room temperature, washed with PBS thrice for 5 min each time, soaked in 0.2% Triton X-100 (Beijing Solarbio Science & Technology, China) for 20 min, and rinsed in PBS thrice for 10 min each time. After that, the samples were blocked in 10% goat serum/PBS for 20 min at 37°C followed by incubation in the mouse anti-vimentin primary antibody (1 : 200 dilution in PBS) at 4°C overnight. After rinsing with PBS, the samples were incubated in FITC-conjugated goat anti-mouse IgG (1 : 50 dilution in PBS) for 2 h at 37°C in the dark room. Finally, after washing with PBS, the samples were dipped in a 4,6-diamidino-2-phenylindole dihydrochloride (DAPI)/PBS solution (Sigma, 3 mg/mL) for 5 min to stain the nuclei (blue fluorescence). The cells were observed under confocal laser scanning microscopy (CLSM, Olympus Fluoview-1000).

### 2.9. Mitochondrial Activity Assay

Mitochondrial activity of the cells was measured using the MTT method at days 1, 5, 10, and 14, respectively. Twenty microliters of 0.5 mg/mL methylthiazolyldiphenyl-tetrazolium bromide (MTT) solution (0.5 mg/mL, Beijing Solarbio Science & Technology, China) was added to each well of the 96-well plate cultures and incubated at 37°C for 4 h in the dark. 150 mL dimethylsulphoxide (DMSO) was subsequently added to each culture to dissolve the purple formazan crystal. The absorbance (OD at 490 nm) was recorded with an ELISA reader (MaxM5, Spectra, USA). The absorbance of the same material in the same solution containing no cells was used as blank reference. Cells cultured on the tissue culture polystyrene (TCPS) were used as the positive reference. Triplicates of each sample were averaged.

### 2.10. Western Blotting

Cells grown on cPU membrane and TCPS 24-well culture plates for 14 d were washed three times with PBS for 5 min each time. Two hundred microliters of radioimmunoprecipitation assay lysis buffer (RIPA) containing phenylmethylsulfonyl fluoride (PMSF) (RIPA : PMSF, 100 : 1, v : v) (Membrane and Cytosol Protein Extraction Kit, Beijing Solarbio Science & Technology, China) was added to each well and kept for 30 min on ice. The cell lysate was collected and centrifuged at 12,000 rpm for 5 min at 4°C. The supernatant was collected in a new microcentrifuge tube. Twenty-five microliters of the supernatant was mixed with 5 *μ*L 5X loading buffer and loaded onto a 12% sodium dodecyl sulfate (SDS) polyacrylamide gel. Electrophoresis was performed in running buffer at 100 V for 2 h. The separated proteins were then transferred onto a polyvinylidene fluoride membrane (PVDF, Roche Diagnostics) at 70 V for 2 h. After blocking with Tris-buffered saline (TBS) containing 5% skim milk for 1 h at room temperature, the membrane was incubated in anti-vimentin mouse monoclonal antibody (1 : 500 dilution in BSA blocking solution) overnight at 4°C. After three rinses with 0.05% Tween-20 in TBS (v/v) (TBS-T), the PVDF membrane was incubated in goat IR Dye 680 anti-mouse IgG (H+L) (1 : 15000 dilution in TBS-T) for 2 h at 37°C. The membrane was then scanned and analyzed using the Odyssey infrared scanning imaging system (Odyssey LI-COR, USA). The gradation of the target band was calculated with the Odyssey infrared scanning imaging system for statistical analysis. Beta-actin was used to normalize the cellular protein content. The results presented were from three separate experiments.

### 2.11. *In Vivo* Biocompatibility

In order to assay the material's* in vivo* biocompatibility, female SD rats (3 months old, 250–300 g) were anesthetized with 5% chloral hydrate (intraperitoneal injection, 6 mg/kg) and implanted subcutaneously with sterilized cPU discs (7 mm in diameter). After a predetermined period of time, the SD rats were sacrificed and samples were explanted with a small amount of surrounding tissue. These tissue specimens were fixed in 10% formalin for 1 h, followed by freeze-embedding and microtome slicing into 4 *μ*m sections. Samples were stained with H&E stain and were analyzed under light microscopy (Olympus CX40, Japan). Images were captured by digital camera (PL-B623CU, Pixelink, Canada).

The animals used in this study were treated in accordance with the ethical committee of Ningbo University and the NIH's Principles of Laboratory Animal Care.

## 3. Results and Discussion

### 3.1. Characterization of PLEG Prepolymer

**Scheme 1:**
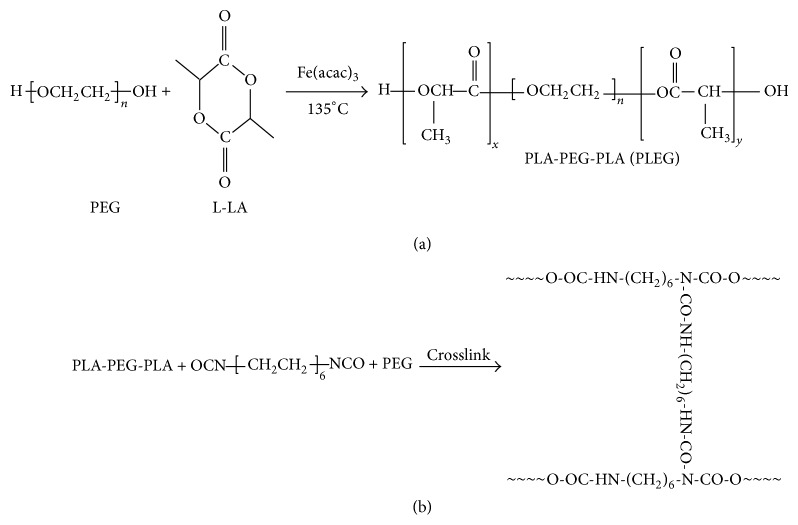
Synthesis of oligomer PLA-PEG-PLA (P) using Fe(acac)_3_ as the catalyst ((a), cited from [16]) and crosslinking of oligomer PLA-PEG-PLA, HDI and PEG (b).

**Figure 1:**
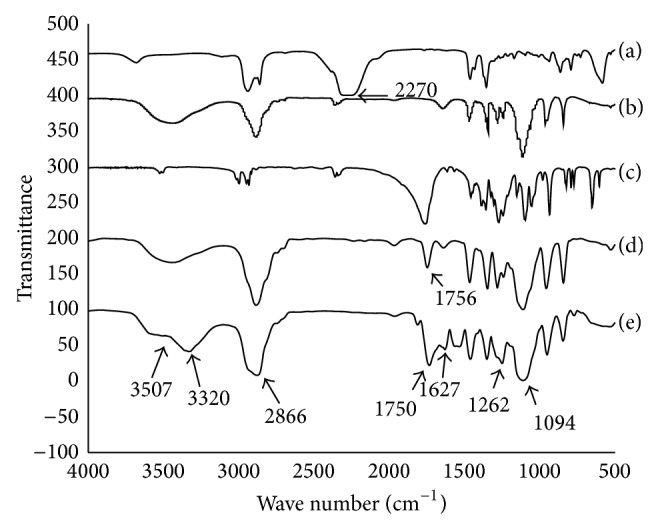
FTIR spectra of HDI (a), PEG (b), L-LA (c), PLEG 4 (d), and cPU (e). The resolution is 4 cm^−1^ and the measuring period is 500–4000 cm^−1^.

The PLEG prepolymer was synthesized from the reaction of L-LA and PEG (molecular weight, 2000 Dalton) using a low toxicity iron compound as the catalyst (Scheme 1(a), [16]). The effect of various ratios of L-LA and PEG on the product's molecular weight was explored (Table 1). The molecular weight of PLEG increased with increasing quantities of L-LA. The molecular weight distribution, 1.16–1.35, is very narrow, which showed the low polydispersity of all products due to effective polymerization. The chemistry of PLEG was verified by FTIR spectra (Figure 1), which showed characteristic spectra for PEG (b), L-LA (c), and PLEG 4 (d). Curve (d) (PLEG) appears to be a sum of curve (b) (PEG) and curve (c) (monomeric L-LA) with a specific peak at 1756 cm^−1^, which can be attributed to ester stretching absorption of L-LA and PLEG. The wide peak at 3450 cm^−1^ can be attributed to hydroxyl groups in the end group of PEG and PLEG (this peak is not present in the L-LA curve). These FTIR characteristics suggest that polymerization occurred between PEG and L-LA to yield the three-block polymer PLEG.

**Figure 2:**
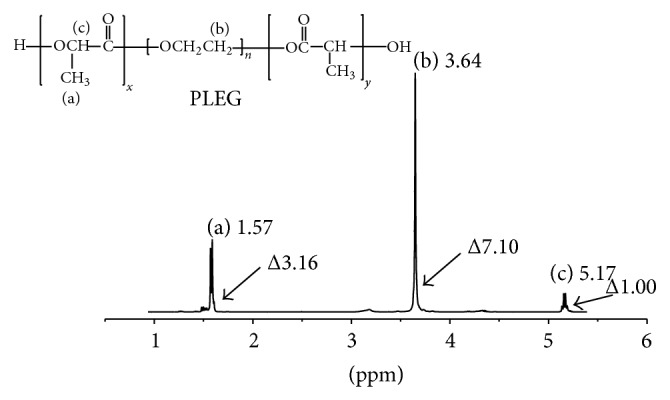
^1^H NMR spectrum of prepolymer PLEG 4.

The reaction was also analyzed by ^1^H NMR using PLEG 4 (mole ratio of PEG : L-LA = 1 : 5) as the experimental sample (Figure 2). Particularly, both protons of the methyl CH_3_ at 1.57 ppm (a) and of the CH with its tertiary carbon attached to hydroxyl and carbonyl groups at 5.17 ppm (c) from the L-LA monomer showed the polylactide (PLA) block, while protons of the CH_2_CH_2_ segment attached to two oxygen atoms at 3.64 ppm (b) were determined to originate from the PEG moiety. The peak area ratio of (a) to (c) was approximately 3 : 1 and confirmed the block structure of -O(CH_3_)CHCO- in PLEG. The peak ratio of (b) to ((a)+(c)), 7.1 : 4.16, is close to 2 : 1, demonstrating that the chains of two PLA subblocks were similar; that is, *x* ≈ *y* in Scheme 1(a). These results indicate that two average blocks of homopolymer PLA and one block of PEG formed the prepolymer PLEG 4 with uniform block weight. This result is considered to be in agreement with the molecular weight measurement of PLEG 4 (6046 Da, Table 1), in which each PEG moiety weighs 2000 Da and each PLA moiety weighs approximately 2000 Da.

In order to reduce the crystallinity of the prepolymer and to obtain a crosslinked polyurethane with good flexibility (low *T*_*g*_), we designed a ring opening reaction between LLA and PEG to produce lower molecular weight prepolymer by using higher amounts of catalyst than those in our previous work [16]. Considering its uniform structure, molecular weight, and polydispersity, PLEG 4 was selected as the reactant for downstream reactions with PEG and HDI.

### 3.2. Chemistry and Properties of cPU

The crosslinking of PLEG 4 and HDI was performed with PEG (Mn 2000) as the extender, to yield the desired cPU (Scheme 1(b)). This crosslinking reaction was confirmed by FTIR spectra (Figure 1), which showed spectra for HDI (a), PEG (b), PLEG (d), and the product cPU (e). Curve (e) (cPU) showed the same peaks as HDI (a), PEG (curve (b)), and PLEG (curve (d)) but without the peak at 2210 cm^−1^, which is a specific peak of the -NCO- group from HDI, which shows that there is no active -NCO- group in the product. Peaks at 3320 and 1627 cm^−1^ in cPU were attributed to C=O stretching absorption in amide groups (O=C-NRR′), while the peak at 3450 cm^−1^ (-OH) was weakened. A peak at 3507 cm^−1^, suggesting N-H stretching, appeared. Peaks at 1750 cm^−1^ (ester stretching absorption), 2866 cm^−1^, and 1094 cm^−1^ (C-H and ether C-O-C stretching absorption originating from PEG) were also observed in the product cPU. Strong absorption from C-H stretching suggested a large amount of PEG component. All these results together imply that crosslinking reactions took place between the reactants HDI, PEG, and PLEG.

**Figure 3:**
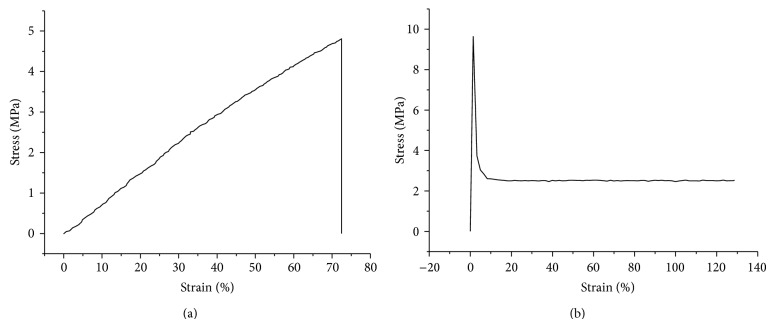
Stress-strain curve of the crosslinked cPU film (a) and larynx tissue (b).

The thermal and mechanical properties of cPU, as well as its wettability and degradation, were evaluated. The curve in Figure 3(a) shows that the glass transition temperature (*T*_*g*_) and melting point (*T*_*m*_) occurred at −22.1°C and 61.5°C. This quite low *T*_*g*_ displayed that cPU was very flexible at body temperature, 37°C. These thermal characteristics meet the mechanical requirements for artificial materials to be used as biomatrix for soft tissues.

The mechanical properties of cPU are shown as a stress strength-strain curve (Figure 3(a)). The maximum strength and ultimate strain were recorded as 4.79 ± 0.76 MPa and 72.5 ± 15.4%. Young's modulus was 31.4 ± 2.8 MPa (Table 2), which was stronger than natural materials such as collagen (0.31 MPa) [17] or chitosan (0.55 MPa) [18] and biodegradable polymers such as polyvinyl pyrrolidone (0.35 MPa) [19] or aloe vera (1.5 MPa) [20], both of which are often used in biomedical applications. However, it is much weaker than the industry poly(ester urethane) (58213 NAT 022), whose maximum strength and ultimate strain are 17 MPa and 900%, respectively [21]. Moreover, the synthesized cPU in our current study is somewhat brittle, as its ultimate strain is 72.5 ± 15.4%. After that, the material broke stone-droppedly. In order to know the mechanics of natural tissues, larynx was tested on the same system with the same parameters. Its maximum strength and ultimate strain was 9.65 ± 0.24 MPa and 1.42 ± 1.31%, respectively. Young's modulus was 692.8 ± 32.1 MPa (Figure 3(b)). The nature of the larynx seems stronger and more rigid than our synthesized cPU. Hypopharynx was supposed to be softer and more flexible than larynx. However, we are short of the detail data due to shortage of the tissue at present.

**Figure 4:**
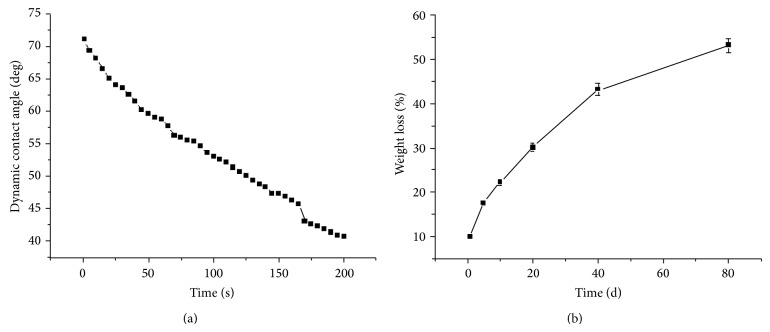
Dynamic contact angle (a) and weight loss (b) of cPU as a function of time. The specimens were immersed in sterilized PBS (pH 7.4) at 37°C and kept in a sealed container during weight loss measurement.

An ideal material for tissue engineering should possess good hydrophilicity and biocompatibility. Thus, a hydrophilic PEG component was introduced. PEG is composed of repeating oxyethylene groups, leading to good hydrophilicity and lipophilicity. It is the most common material used to improve hydrophilicity in a scaffold and further promotes the biocompatability and cytocompatibility of the scaffold [22, 23]. In our case, PEG was introduced as a component of our material. The hydrophilicity of the cPU was evaluated via static and dynamic contact angle measurements. The instant water contact angle was measured as 71.1 ± 1.4° (Table 2). This value decreased rapidly with time (Figure 4(a)). After 200 s, the water drop disappeared, presumably absorbed by the material. We also measured the maximum water uptake, 229.7 ± 18.7% (Table 2), after dipping the material in water for 10 h, at which point it became a hydrogel with good flexibility and softness, which are good characteristics for hypopharyngeal tissue engineering. An important consideration when designing scaffolds for tissue engineering is degradation, as the rate of degradation of the scaffold should ideally match the growth rate of cells and tissues. For biodegradable polymers, degradation usually takes place in 4 steps: water absorption, reduction of mechanical properties (modulus & strength), reduction of molar mass, and weight loss due to diffusion of soluble oligomeric components [24, 25]. Therefore, we tested the scaffold's degradation by weight loss measurements. Our measurement of* in vitro *degradation may help us understand and predict the* in vivo* behavior of these scaffolds. The* in vitro* degradation test was performed over a span of 80 d (Figure 4(b)) and cPU showed rapid degradation kinetics. Weight loss reached 30% after the first 20 d and up to 52.5% by day 80, which was faster than other materials such as PLLA and PCL [26–28]. It is likely that the good wettability and high water uptake greatly promote the material's degradation.

### 3.3. *In Vitro* Cytocompatibility and* In Vivo* Biocompatibility

**Figure 5:**
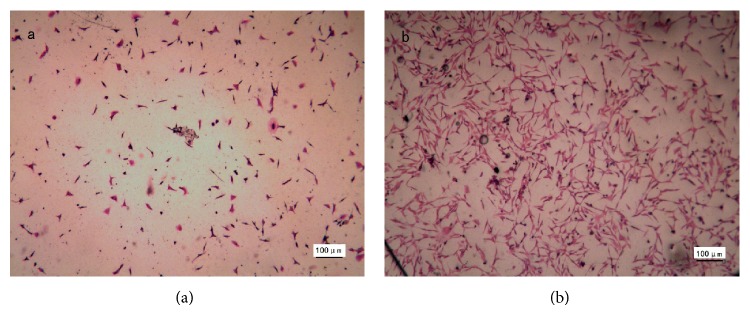
Light microscopy images of cells cultured on cPU membrane for 2 d (a) and 14 d (b). Cells were stained by H&E. The seeding density was 4 × 10^4^ cells/mL. Scale bar 100 *μ*m.

**Figure 6:**
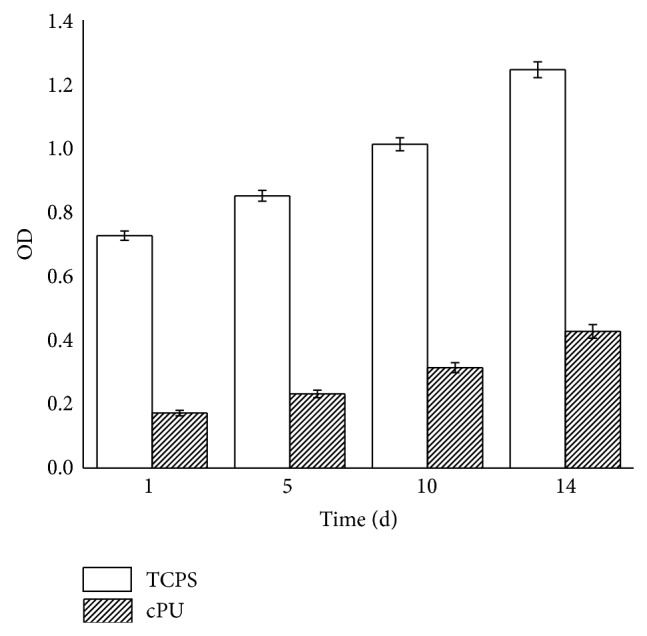
Mitochondrial activity as shown by absorbance at 490 nm. Cells were seeded at a density of 4 × 10^4^ cells/mL and cultured for 1 day, 5 days, 10 days, and 14 days.

**Figure 7:**
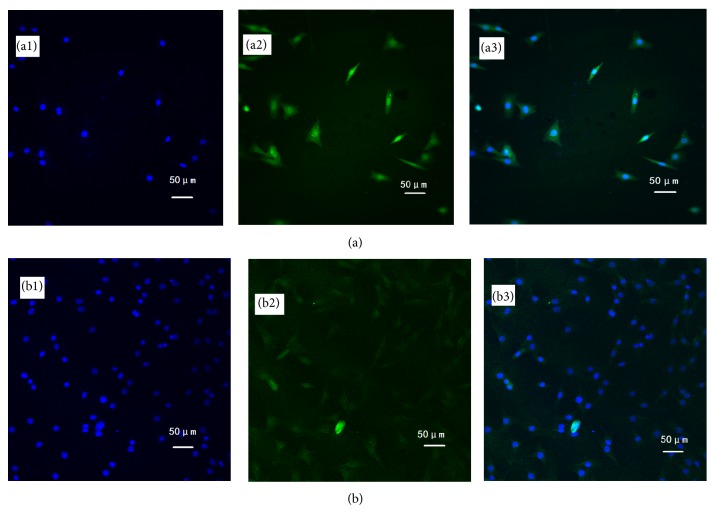
Immunofluorescence of cells cultured on cPU membrane for 2 d (a) and 14 d (b). Anti-vimentin was used as the primary antibody and appears in the cytoplasm as green fluorescence. The nucleus is blue from DAPI staining. The seeding density was 4 × 10^4^ cells/mL. Image (3) is an overlay of images (1) and (2). Scale bar 50 *μ*m.

**Figure 8:**
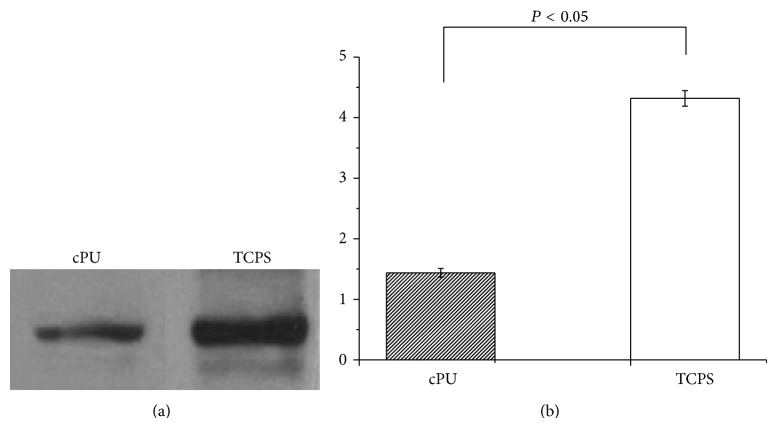
Vimentin expression by cells seeded on cPU and TCPS for 14 d, analyzed by Western blotting. (a) Strips of cPU and TCPS. (b) Quantitative analysis of vimentin expression. Beta-actin was used as the reference to normalize the cellular protein content. *P* value is <0.05.

The cytocompatibility of the synthesized cPU was firstly measured via human hypopharyngeal fibroblast seeding. Cell morphology, activity, phenotype, and protein expression were tested. Due to its hydrophilicity, cPU swelled significantly in the cell culture medium and in the body during the* in vivo* test. As a result, scanning electron microscopy (SEM) was not used for the observation of cell morphology. Under light microscopy, cells were visible after primary fibroblasts were cultured on the cPU membrane for 2 d (Figure 5(a)). There were many more live cells on the membrane at day 14 (Figure 5(b)). Cells were stained by H&E in order to visualize them on the membrane. MTT test showed that the seeded cells grew and proliferated with time, although the mitochondrial activity (as determined by OD value) was lower than that on TCPS at every time point during the 14 d culture span (Figure 6). Mitochondrial activity on cPU at day 14 only reached 36% of that on TCPS. This value was similar to the activity of skeletal muscle cell from human hypopharynx on poly(ester urethane) (58213 NAT 022, China) [21]. The much higher hydrophicility of cPU resulted in the lowered cell adhesion (OD at day 1). However, the increase rate of OD value from day 1 to day 14 is quite similar between cPU and TCPS, which means the similar cell proliferation capability between these two matrices. On the other hand, cPU was found to possess rapid degradation rate from weight loss test (Figure 4(b)). It may make fibroblasts very difficult to adhere firmly on the matrix with the passing time, leading to lower test results than the reality is. Despite this, the cells cultured on cPU had a fibroblast-like phenotype. Vimentin is the intermediate filament in fibroblasts. Thus, it is a reliable fibroblast marker. Using anti-vimentin as the primary antibody, cells on cPU substrates were immuno-stained (green fluorescence) to confirm the fibroblasts after they had been cultured* in vitro* from 2 d to 14 d (Figure 7). Quantification of vimentin expression verified that the cultured cells still had the ability to differentiate (Figure 8). The amount of vimentin secreted by cells grown on cPU was 35% of that of cells on TCPS at day 14, which was in agreement with the result of mitochondrial activity analysis.

**Figure 9:**
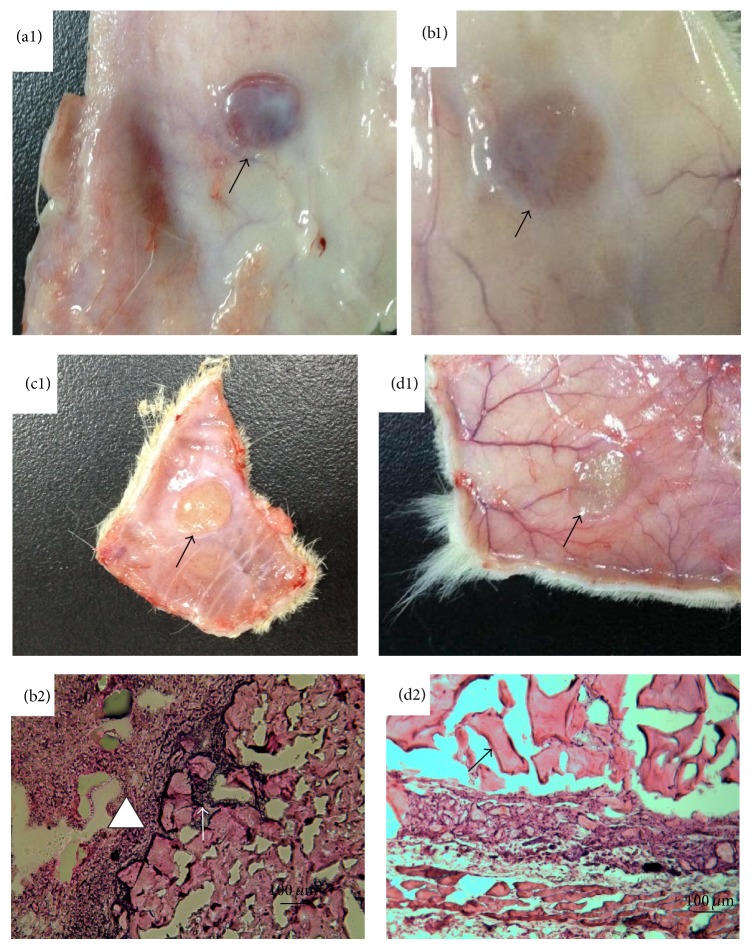
Appearance of subcutaneously implanted cPU membrane in the back of SD rats for 7 d (a1), 21 d (b1), 49 d (c1), and 70 d (d1); arrow indicates the location of the material. (b2) H&E staining of microtome section after 21 d implantation; white triangle and arrow indicate the site filled with inflammatory cells. (d2) H&E staining of microtome section after 70 d implantation. Arrow indicates the biodegraded fragment. Scale bar 100 *μ*m.

In order to ascertain the* in vivo* biocompatibility of our synthetic material, cPU was implanted subcutaneously into the back of SD rats. After implantation for 7, 21, 49, and 70 d, SD rats were anaesthetized and the location of the cPU membrane was exposed. By visual observation, the subcutaneous samples were already enclosed in the skin hypodermis after 7 d. However, a small amount of inflammation and serious blood swelling were observed all around the membrane (Figure 9(a1), arrow). By day 49, the blood swelling was comparatively reduced (Figure 9(b1), arrow). However, many inflammatory cells filled the circumference of the material (Figure 9(b2), white triangle), while some inflammatory cells had infiltrated into the material interspace (Figure 9(b2), white arrow). The inflammation and blood swelling reduced significantly by day 49 (Figure 9(c1), arrow) and were completely resolved by day 70 (Figure 9(d1), arrow). Angiogenesis was observed around the sample, which greatly promotes the biocompatibility of the scaffold. H&E staining revealed the clear tissue structure (Figure 9(d2)). However, during this period of time, the material had begun to biodegrade and some fragments could be observed (Figure 9(d2), arrow). We considered the material to have been accepted by the animal body.

## 4. Conclusions

A biodegradable polyurethane cPU was produced by crosslinking HDI, PEG, and prepolymer PLEG, which was synthesized from the reaction between L-LA and PEG using Fe(acac)_3_ as the catalyst. The reaction chemistry was followed and analyzed via FTIR spectroscopy, ^1^H NMR, and GPC. The synthesized cPU possesses low *T*_*g*_ (−22°C), good hydrophilicity, and relatively fast degradation. It also has the ability to support the growth of human hypopharyngeal fibroblasts. Subcutaneous implantation in SD rats suggests that the material has good biocompatibility. However, the material is still weak and degrades rather quickly when compared with industrial polyurethane products. The synthesis reaction and product chemistry are being improved in our laboratory in order to meet the requirements of a good scaffolding material for hypopharyngeal tissue engineering.

## Figures and Tables

**Table 1:** The component feeding ratios and GPC measurements of prepolymer PLEG.

Copolymer	PEG/L-LA in feeding (mol)	*M*_*n*_ (Da)	*M*_*w*_ (Da)	*M*_*w*_/*M*_*n*_
PLEG 1	1.2 : 1	4365	5926	1.3576
PLEG 2	1 : 1	4702	5977	1.2709
PLEG 3	1 : 2	5422	6311	1.1638
PLEG 4	1 : 5	6046	7312	1.2090

**Table 2:** Properties of cPU film. The cPU was prepared by crosslinking of PLEG4, PEG, and HDI (mole ratio, 1.65 : 10 : 2.9).

*T*_*g*_ (°C)	*T*_*m*_ (°C)	*Ó* (MPa)	*ε* (%)	*E* (MPa)	Static contact angle (°)	Water uptake (%)
−22.1 ± 2.4	61.5 ± 3.5	4.79 ± 0.76	72.5 ± 15.4	31.4 ± 2.8	71.1 ± 1.4	229.7 ± 18.7
